# Predictors of exceeding emergency under-five mortality thresholds using small-scale survey data from humanitarian settings (1999 – 2020): considerations for measles vaccination, malnutrition, and displacement status

**DOI:** 10.1186/s13690-022-00916-0

**Published:** 2022-06-28

**Authors:** Thomas Jideofor Ogbu, Sarah Elizabeth Scales, Maria Moitinho de Almeida, Joris Adriaan Frank van Loenhout, Niko Speybroeck, Debarati Guha-Sapir

**Affiliations:** 1 Centre for Research On the Epidemiology of Disasters (CRED), Institute of Health and Society, Université Catholique de Louvain, Brussels, Belgium; 2 University of Delaware College of Health Science, Program in Epidemiology, Newark, Delaware USA

**Keywords:** Under-five mortality, Emergency threshold, Measles-containing vaccine, Global acute malnutrition, Internally displaced, Refugees, Affected residents

## Abstract

**Background:**

Quantifying the effect of measles containing vaccine (MCV) coverage and the prevalence of global acute malnutrition (GAM) on mortality levels in populations of displaced and crisis-affected resident children is important for intervention programming in humanitarian emergencies.

**Methods:**

A total of 1597 surveys containing data on under-five death rate, population status (internally displaced, refugee, or crisis-affected resident), measles containing vaccine coverage, and global acute malnutrition were extracted from the Complex Emergency Database (CE-DAT). Under-five mortality rates were dichotomized to those exceeding critical levels or otherwise. A Bayesian multivariable mixed-effect logistic regression model was used to assess the association between an under-five death rate (U5DR) exceeding this threshold and population status (i.e., internally displaced, refugees or residents), GAM prevalence (proxy for food security), and MCV coverage.

**Results:**

The prevalence of GAM, MCV and U5DR were higher in internally displaced children (IDC) with values of 14.6%, 69.9% and 2.07 deaths per 10 000 per day, respectively. Refugee populations had lower average under-five mortality rate (0.89 deaths per 10 000 per day), GAM of 12.0% and the highest measles containing vaccine coverage (80.0%). In crisis-affected residents the prevalence of GAM, MCV and average U5DR are 11.1%, 65.5% and 1.20 deaths per 10 000 per day respectively. In mixed-effect logistic model taking 2 deaths per 10 000 children less than five years old per as emergency threshold (Model III); MCV (AOR = 0.66, 95% Highest Density Interval (HDI): 0.57, 0.78), GAM (AOR = 1.79, 95% HDI: 1.52, 2.12) were associated with a reduction of the odds of U5DR exceeding critical level accounting for country-specific levels of variability. The odds of U5DR exceeding critical level (2/10000/day) in crisis-affected resident children and refugees were 0.36 (95% HDI: 0.22, 0.58) and 0.25(95% HDI: 0.11, 0.55) less than amongst IDP children adjusting for GAM and MCV. In considering country specific yearly median U5DR (model IV) the odds of U5DR exceeding twice the median U5DR were associated with MCV (AOR = 0.72, 95% HDI: 0.64, 0.82), GAM (AOR = 1.53, 95%HDI: 1.34, 1.76). The odds of U5DR exceeding critical level in crisis-affected resident children was 0.30(95% HDI: 0.20, 0.45) less than IDP children, after adjusting for MCV and GAM. We found no difference between the odds of U5DR exceeding twice the country level median U5DR in the refugee population compared to the IDPs.

**Conclusions:**

In this study vaccination coverage and global acute malnutrition (proxy for food security) were associated with U5DR exceeding critical level. The emergency threshold for IDPs and affected residents is significantly different and consistent across the different outcomes, whereas the result is inconsistent for IDPs and refugees. Continued improvement in measles vaccination coverage and reduction of malnutrition in humanitarian emergencies have the potential to minimize the deterioration of mortality level amongst children in emergency settings. To generate a robust understanding of the critical level of child mortality in displaced and affected resident populations, studies accounting for the impact of the duration of displacement, contextual factors in humanitarian settings, and the level of humanitarian assistance provided are needed.

**Supplementary Information:**

The online version contains supplementary material available at 10.1186/s13690-022-00916-0.

## Background

Worldwide more than 1 billion people live in countries confronted with either new, existing or resurgent crises, and more than 50% of those people live in poverty [1]. Millions of people have been forced to flee their homes because of protracted violence and conflicts, with children bearing a disproportionate share of the burden. Globally, the number of forcibly displaced people increased from 79.5 million people at the end of 2019 to 82.4 million people at the end of 2020 [2, 3]. Regionally, Africa houses the highest number of countries experiencing forcible displacement and more than 67% of displacement persons abroad are from five countries — Syrian Arab Republic, Venezuela, Afghanistan, South Sudan and Myanmar [2, 3].

Refugees are often displaced due to violent conflicts and are susceptible to poor health status. Unlike internally displaced persons (IDPs), refugees are displaced persons that cross internationally recognized borders, have rights under International Humanitarian Law, and are protected by International Refugee Law [4]. In addition to the displaced persons, affected residents (individuals, groups, communities or countries that are directly or indirectly affected by humanitarian crisis) also experience the impacts of humanitarian crises and share the harsh realities of conflicts and violence, whether internal or in neighboring states [5]. Specifically, due to violence, conflicts, and disasters, more than 12 million children are refugees and more than 21 million are internally displaced at any given point in a given year [6].

The effects of humanitarian emergencies on the health status of the displaced and affected residents are not the same. For instance, a study in Somalia suggested that IDPs have higher risks of mortality than affected residents [7]. A comprehensive study based on multiple surveys conducted in humanitarian emergency settings suggested that IDPs have higher excess overall mortality than affected resident and refugee populations [8]. In 2018, across five refugee camps in Ethiopia, Somali refugees reported a lower average under-five mortality than values reported in 2019 for affected Somali residents of Hudur in the Bakool region [9, 10]. A 2005 retrospective survey conducted in Darfur revealed that under-five death rate (U5DR) and malnutrition were higher in IDPs than in resident populations in the North and West regions of Darfur [11].

The prevalence of malnutrition in humanitarian settings is usually high, compounding vulnerabilities resulting from insecurity, destruction of infrastructure, and limited access to civil and health services [12–19]. Together, high prevalence of malnutrition, low immunization coverage, and commensurate increases in the incidence of vaccine preventable communicable diseases foment high-mortality conditions in humanitarian settings [20–27]. For example, measles, one of the leading causes of child death in humanitarian emergencies, is more likely to have severe sequelae and higher mortality rates if infected children are under or malnourished [28]. Because the distribution of healthcare conditions and needs varies across populations, it is critical to understand how different health needs – such as food security or routine immunization – help explain the drivers of differential mortality profiles among affected residents versus displaced populations [29]. During a measles outbreak in refugee camps Somalia and Tanzania, for instance, disparate underlying vaccination coverage between new arrivals and the affected resident populations helped prime conditions for such outbreaks [30, 31].

Age-specific U5DR is one of the commonly used indicators for measuring the severity of a crisis, the volume of aid required by the affected population, and the evaluation of humanitarian response efforts. Typically, mortality thresholds indicate if the affected population’s condition has reached a critical level. For U5DR, according to the Humanitarian Charter and Minimum Standard, the doubling of the baseline mortality rate is considered an emergency threshold [32]. In the absence of a known baseline mortality value or when that measure is in doubt, a U5DR above 2/10 000/day is an indication of public health concern [32]. Humanitarian agencies use emergency thresholds to decide whether to continue, reduce, or commit more resources to their programs. However, Weissman has extensively addressed the limitations of the recommended and often contentious use of threshold value [33].

In this study, we evaluate variations in the association between the odds of U5DR exceeding emergency threshold in forcibly displaced and affected resident population using data from complex emergencies. Nutrition and vaccination coverage are included as moderating factors, with GAM and Measles Containing Vaccination (MCV) coverage – two commonly reported indicators – serving as a proxies for food security and vaccination coverage, respectively [34].

## Methods

### Data sources

We extracted aggregated survey data on under-five death rates (all-cause mortality in children less than five years old), GAM, MCV, and population status (IDPs, refugees and crisis affected residents) from the Complex Emergence Database (CE-DAT). CE-DAT is a repository of health, nutrition and mortality indicators from survey reports from complex emergency settings conducted and contributed by humanitarian organizations. The CE-DAT database contains 3432 surveys from 58 countries and territories. Nutrition, morbidity and mortality estimates (Crude Death Rate and U5DR) are extracted from retrospective household surveys conducted by humanitarian agencies and subsequently entered into and stored in CE-DAT. The estimation of the reported death rates is based on information such as recall period, number of deaths, number of births and number of people living or joining the household within a given period. Details of CE-DAT database are discussed elsewhere [35]. We also extracted country level under-five mortality rate per 1000 live deaths (U5MR) from United Nations International Children’s Emergency Fund database.

### Selection criteria

**Fig. 1:**
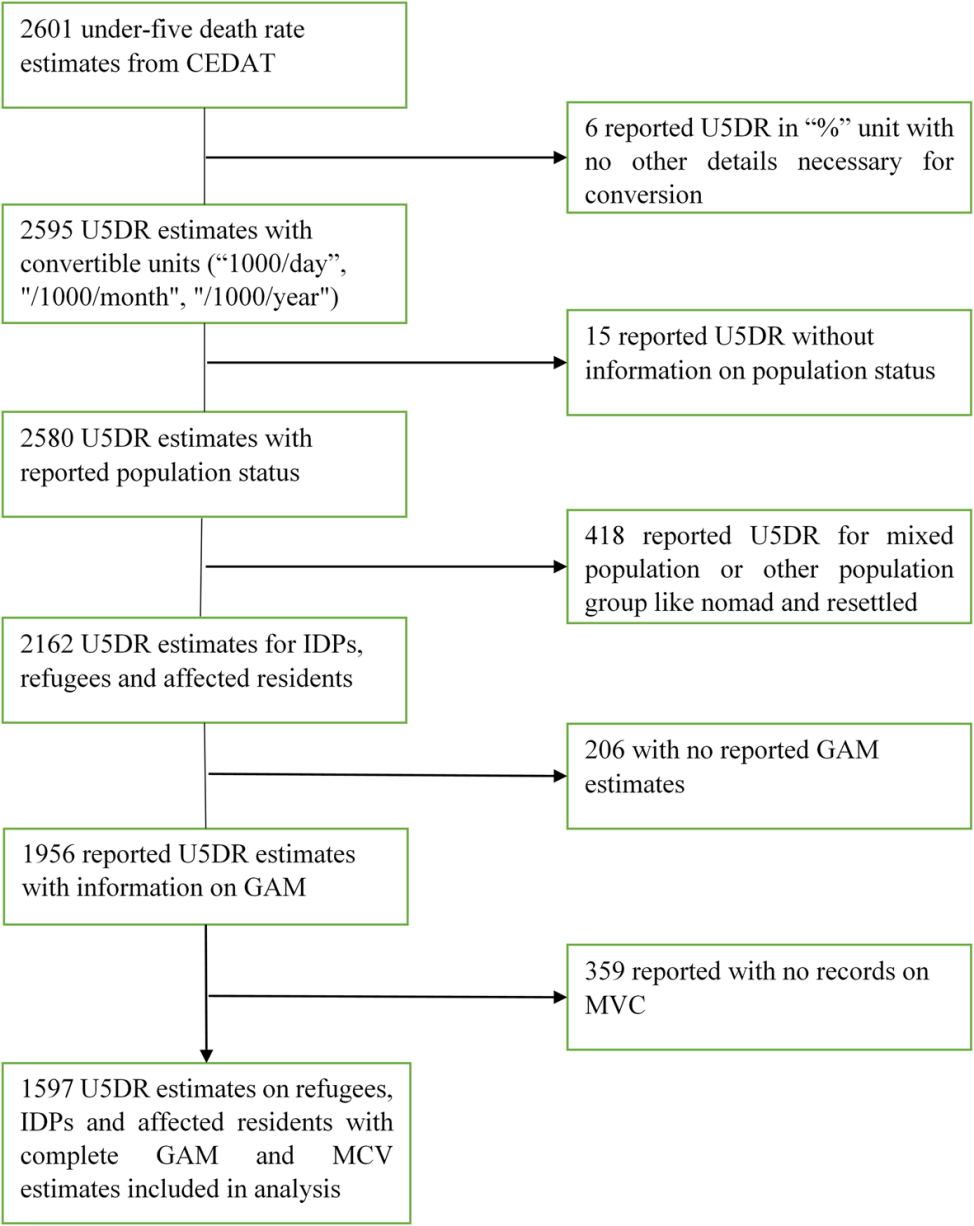
selection of 1579 estimates used in our analysis

We included survey data that contains complete information on U5DR, GAM, MCV and status of affected-population. The population status had to be either of IDPs, refugees or crisis-affected residents. Finally, U5DR had to be either reported in units of 10,000 population/day or in units that are convertible to 10,000 population/day. Figure 1 shows the selection of the 1597 estimates from 37 countries covering the period 1999 to 2020 used this study.

### Variables

In addition to the U5DR data collected from CE-DAT, we collected UNICEF-reported, country-level yearly median under-five morality rate (U5MR) from 1999 to 2020 [36]. We obtained the yearly median under-five death rate (*U5DRmd*) by approximating the U5MR as follows:

$$\frac{10000*U5MR}{1000*5*365.25} \approx U5DRmd$$

We have two independent outcomes. The first outcome variable was dichotomised and values corresponding to U5DRs below 2 deaths/10 000 population/day were set as reference group and the second outcome variable was dichotomised such that values of U5DRs below twice the value of the yearly median U5DR were set as the reference group. The first outcome was used in Model I & III and the second outcome in Model II & IV. In both models the independent variables are GAM, MCV, and population type. GAM was measured as the percentage of children between 6 and 59 months old classified as having low weight-for-height and/or oedema. We used GAM as a more distant indicator of the food security situation and as a proxy of nutritional status in a population, GAM is important to consider because malnutrition increases the risks of illness and death amongst children, and serves as an indication of food security in humanitarian emergencies [37]. We assumed that the level of malnutrition serves as an indication of food security. However, this may not be a perfect representation of food security because GAM could change within a short period. MCV measures the percentage of children aged 9—59 months who received at least one dose of measles-containing vaccine by their second birthday. As a routine, universally indicated, early childhood vaccine, MCV coverage is an indication of the level of access to (or disruption and therefore lack of) routine healthcare services [38]. The population status refers here to a categorical variable with three groups: IDP, resident (crisis-affected residents), and refugee.

### Statistical analyses

We assessed the relationship between the odds of U5DR exceeding emergency threshold and population status, MCV and GAM using a Bayesian logistics fixed and mixed-effect model. To examine how country specific thresholds may have influenced our estimates, we fitted a second model to assess the relationship between the odds of U5DR exceeding twice the reported median U5DR and population status, MCV and GAM. The standard linear fixed-effect model assumes that observations are independent. Given that observations collected within the same country may be correlated (underlying humanitarian emergencies within a country may have the same root cause and populations more related), intra-country correlation was accounted for when analyzing the data. The advantage of the linear mixed-effect model, including fixed and random effects, is that observations within the same country are assumed to be correlated by a random effect parameter, which allows the quantification of the between and within-country variability [39]. The advantage of the Bayesian technique over the frequentist approach is that information from past results, if available, can be incorporated via priors improving the model, and complex models with a larger number of random variance components can be easily fit and interpreted [40, 41].

Accordingly, we fitted a fixed effect model (Model I) using the first outcome (U5DR < 2/10000/day as reference), with GAM, MCV, an interaction term between GAM and MCV, and population status as the independent variables, while assigning a non-informative prior to the model parameters. Then, we extended the fixed-effect model by including a random term for country level estimate, based on the assumption that the random effect term follows a normal distribution with mean of 0 and variance tau (Eq. 2), to obtain a mixed-effect Model III. To assess if the association observed in our analysis could be explained by the difference in the mortality profile of the different countries, we refitted the fixed-effect model (Model II) and mixed-effect model (Model IV) using the second outcome variable ( U5DR < 2*U5DRmd). The interaction term was not statistically significant in the fixed and mixed-effect models; hence, we excluded the interaction term, and our final models contain GAM, MCV and population status. Given that the mixed-effect models capture possible country variability we interpreted the results of the mixed-effect models.

$${y}_{ik} \sim dbern({mu}_{i})$$

1 $$logit\left({mu}_{i}\right)= \alpha + \sum_{j=1}^{5}{\beta }_{j}{X}_{j}$$

2 $$logit\left({mu}_{i}\right)= \alpha + \sum_{j=1}^{5}{\beta }_{j}{X}_{j}+ {\eta }_{k}$$

where $i=1, \dots , n$, $n$ is the number of observations,

$k$= 1 … $m$ is the number of countries, ${y}_{ij}$ is the $ith$ response in country k,

$\alpha$ is the intercept, ${\beta }_{j}$ are regression coefficients for the independent variables ${X}_{j}$ (GAM, MVC, resident and refugee), $\alpha$ and ${\beta }_{j} \sim dnorm(0, 0.001)$, ${\eta }_{k}$ (random effect term) $\sim dnorm(0,{\tau }^{2})$.

We assigned non-informative inverse gamma prior to $\tau , ie {\tau }^{2} \sim Invgamma(0.001, 0.001)$.

We used the Markov Chain Monte Carlo (MCMC) sampling procedure to estimate the model parameters for our model using three MCMC chains with different starting values. The results obtained were derived by using a burn-in period of 50 000 iterations, with a thin rate of taking one point for every 10 iterations to account for possible autocorrelation. The Geweke diagnostic and Brooks-Gelman-Rubin diagnostics were considered for checking stationarity of chains, and we assessed our model fit using the posterior predictive check (PPC) and Bayesian p-value [42]. Statistical analyses were performed using RStudio version 4.0.2, R2jags package, version 0.6.1 [43], HDInterval package, version 0.2.2 [44] and ggplot2 package, version 3.3.2 [45] for graphics. The full reproducible codes are available in supplementary material (additional file 1).

### Model analyses

We fitted fixed and mixed-effect models. Visual inspection of the traceplots indicated convergence with a Gelma-Rubin “R-HAT” value of 1 and a good mix of the three chains (additional file 3). The Bayesian *p*-value of the posterior predictive check were 0.53 (Model I) and 0.54 (Model II) for the fixed effects models, and 0.59 (Model III) and 0.4 (model IV). For the mixed-effect models, these values are closer to 0.5 than to 0 or 1 indicating an appropriate fit [42]. The PPC plots show considerable fit (Supplementary additional file 4 and additional file 5).

## Results

### Descriptive statistics

Of the 1597 surveys included, 476 (29.8%) reported the number of children sampled, giving a total sample size of 493 952 children.

All the data were obtained from humanitarian emergency settings and/or low-income countries. The majority of the surveys (91.3%) were from Africa as shown in Table 1 below.

**Table 1:** Distribution of selected surveys by continents and population status

Continent	Resident	IDPs	Refugee	Total
Africa	1114	181	164	1459
Asia	78	5	13	96
North America	41	0	0	41
South America	1	0	0	1

A total of 557 841 households were samples across 661 surveys (41.4%). The remaining surveys (28.8%) reported neither number of children nor household sample. Summary statistics of the U5DR, GAM, and MCV are presented in Table 2.

**Table 2:** Description of data used in the study

Population status	Country N	Obs N	U5DR (10,000 per day) mean(SD)	Prevalence of GAM (%) mean(SD)	Prevalence of MCV (%) mean(SD)
Resident	34	1234	1.20 (1.46)	11.10 (6.70)	65.50 (23.70)
IDP	11	186	2.07 (2.28)	14.6 0 (8.60)	69.90 (22.10)
Refugee	13	177	0.89 (0.74)	12.0 0 (7.30)	80.0 (20.50)

The full distribution of the data by country and year are presented in supplementary material (additional file 2).

### Predictors of odds of under-five death rate exceeding emergency threshold

The estimates from the fixed-effect models are similar to results of the mixed-effect models. However, since the mixed-effect model has the advantage of considering variability within country we decided to interpret the results of the mixed-effect models instead. The results of the fixed-effect model are shown in Table 3 below.

**Table 3:** Odds ratios, 95% High density interval of Multivariable fixed-effect analysis of determinants of under-five death rates exceeding critical level in humanitarian emergency settings

**Variable**	**Model I**^b^	**Model II**^b^
	Est[95%HDI]	Est[95%HDI]
MCV	0.76[0.66, 0.88]*	0.85[0.76, 0.95]*
GAM	1.48[1.30, 1.69]*	1.48[1.32, 1.66]*
**IDP**	**1**	**1**
Refugee	0.17[0.08, 0.33]*	0.47[0.30, 0.72]*
Resident	0.36[0.25, 0.52]*	0.37[0.27, 0.51]*

^a^Odds ratios of the Bayesian logistics fixed-effect models

^b^Same sample size (*n* = 1597) was used for both Model I & II. Model I the critical value for U5DR is 2/10 000/day and in Model B the critical U5DR value is double country level reported U5DR for a given period

^*^statistical significant at alpha = 0.05 level

### The consequence of forced displacement on the odds of U5DR exceeding emergency threshold in humanitarian settings

The mixed-effect models reveal a country-specific U5DR with a standard error from zero of 1.18 and 0.9 for model III and IV respectively. Based on our data, model III and IV revealed that a unit (1%) increment in vaccination coverage leads to a reduction of the odds of U5DR exceeding emergency thresholds in IDPs by approximately 33.6% (AR = 0.66, 95% HDI: 0.57, 0.78) and 28.8% (AR = 0.72, 95% HDI: 0.64, 0.82), respectively, when country variability is accounted for and GAM is constant. Assuming that the population status is constant, if the level of GAM increases by 1 unit, we expect a higher odds of 79% (AR = 1.79, 95% HDI: 1.52, 2.12) and 53% (AR = 1.53, 95% HDI: 1.34, 1.76) of U5DR exceeding emergency threshold in IDPs when country variability is considered.

Assuming that MCV and GAM are the same across the different populations (IDPs, affected residents, and refugees), the odds of U5DR exceeding emergency thresholds for refugees is 75.0% lower in model I (AR = 0.25, 95% HDI: 0.11, 0.55) and 28.0% lower in model III (AR = 0.82, 95% HDI: 0.47, 1.44) than the odds of exceeding the emergency threshold values by IDPs. While for affected residents, the odds of U5DR exceeding thresholds in model III is 64.0% lower in model III (AR = 0.36, 95% HDI: 0.23, 0.58) and 70.0% lower in model IV (AR = 0.30, 95%HDI: 0.20, 0.45) than the odds of IDPs exceeding the emergency threshold.

The results of the mixed-effect analyses are shown in Table 4 and for the random intercept estimates (see supplementary material additional file 6).

**Table 4:** Odds ratios, 95% High density interval of Multivariable mixed effect analysis of determinants of under-five death rates exceeding critical level in humanitarian emergency settings

**Variable**	**Model III**^a^	**Model IV**^a^
	OR^b^ [95%HDI]	OR^b^ [95%HDI]
MCV	0.66[0.57, 0.78]*	0.72[0.64, 0.82]*
GAM	1.79[1.52, 2.12]*	1.53[1.34, 1.76]*
**IDP**	**1**	**1**
Refugee	0.25[0.11, 0.55]*	0.82[0.47, 1.44]
Resident	0.36[0.23, 0.58]*	0.30[0.20, 0.45]*

^a^Adjusted odds ratio of the posterior mean estimates of Bayesian mixed effect logistics analysis after taking into account data and priors

^b^Same sample size (*n* = 1597) was used for both models. Model III the critical value for U5DR is 2/10 000/day and in Model IV the critical U5DR value is double country level reported U5DR for a given period

^*^statistical significant at alpha = 0.05 level

## Discussion

With an increase in forced displacement, more children are significantly affected, especially considering that displacement has continued to increase over a number of years [2]. Poor nutritional status and the lack of essential vaccinations has worsened the mortality risk of forcibly displaced children. In this study, we assessed the difference in the odds of under-five mortality exceeding critical levels amongst children in IDP, refugee, and affected resident populations. The strength of our study is the use of several retrospective surveys from various countries with different complex emergency situations, allowing us to compare the critical level of U5DR in internally displaced populations with that of crisis-affected residents and refugees.

We found that the odds of under-five mortality exceeding critical level for children in affected resident and refugee populations (The difference between refugees and internally displaced persons is not consistently greater than zero across all models) are lower than that of children in IDP populations when accounting for nutrition and vaccination coverage. This finding is in concordance with findings from retrospective mortality surveys conducted in Darfur [11]. A possible explanation is that in humanitarian emergencies, people who are most at risk are most likely to locate to a safer place. Since the mass displacement of people results in temporary, mostly crowded living arrangements that are characterized by inadequate sanitation facilities and poor hygiene [19], IDPs are likely to present with comparatively poor health status to the non-displaced affected resident. IDPs are often more difficult for humanitarian agencies to locate and reach, unlike refugees and affected resident populations. This makes it increasingly difficult to provide life-saving aid and healthcare services to them, hence necessitating unique considerations for care [46].

In situations where displaced persons are located and cared for, their presence may increase the demands for goods and services, potentially negatively affecting the provision of these goods and services to residents [47–49].

Furthermore, the results of our analyses are similar to studies that showed that in humanitarian crises, improving food security [13, 50, 51] and increasing vaccination coverage [52–54] are linked with the reduction of child mortality. Addressing the issues of displacement and the resultant effect on the health status of affected children via humanitarian activities [55] provides a temporary solution. To achieve a long-term permanent solution requires infrastructural, social, and political stability, although these are often difficult to achieve due to the complex nature of conflict environments.

### Limitations

This study has some limitations. The data used were contributed freely to the CE-DAT project by NGOs without obligation. This might lead to information bias, such that certain surveys are overrepresented while others (with negative findings) are under-represented. Secondly, the database has not been updated systematically in the last years due to lack of sustained funding. The implication is that the most recent studies were under-represented in our data. For many of the estimates there is not enough information (e.g., sample sizes, design effects, number of child deaths, precise affected population and context specific information) required for detailed analysis. As in most studies, the survey results are prone to recall bias. We have no way to quantify the scale of potential bias and its associated effect on our results. Although the use of the recommended threshold by the Humanitarian Charter and Minimum Standard is controversial in some contexts, we feel that it represents the best available estimate. However, lack of more granular, emergency-specific country/region/ecological and zone/district level mortality thresholds may have masked the true variations of our estimates. The classification of population into IDPs and refugee does not provide further information on how long the population has been displaced. A previous study has indicated that duration of displacement is an important variable to consider when studying U5DR in these groups [56]. Future studies should quantify and assess the impacts of duration of displacement, as displaced households or refugees may have stayed long enough to share similar health status as the affected residence.

## Conclusion

Notwithstanding the aforementioned limitations, we believe that this study provides insight into important factors that determine critical under-five mortality level in humanitarian emergencies, such as displacement status, measles vaccination coverage and prevalence of global acute malnutrition. In this study, U5DR levels over the critical level were linked to vaccination coverage and global acute malnutrition. The emergency threshold is considerably different and consistent across the different outcomes for IDPs and affected residents, whereas the conclusion is inconsistent for IDPs and refugees. Understandably, ending forced displacement is a difficult and tedious process that requires coordinated humanitarian efforts and political commitment to address the root causes. Nevertheless, deliberate efforts to not only locate forceful displaced and crises affected children but also to improve access to quality healthcare and food security in humanitarian emergencies, may significantly reduce mortality measures below critical levels. Furthermore, there is need for studies that consider additional elements in their analysis, such as the impact of the duration of displacement, the humanitarian contexts, and levels of interventions provided in order to inform a broader understanding of the disparities in the critical level of child mortality amongst IDPs, refugees, and affected-residents.

## Supplementary Information

**Additional file 1.** R code for Bayesian multivariable Fixed and Mixed-Effect logistic regression.

**Additional file 2.** Summary of the surveys (n=1597) from 1999 to 2020.

**Additional file 3:** **Figure 1.** Traceplots of mixed-effect model III.** Figure 2.** Traceplots of mixed-effect model IV.

**Additional file 4.** PPC check for Bayesian multivariable mixed-effect logistic regression model.

**Additional file 5.** Posterior predictive check for Bayesian multivariable fixed-effect logistic regression model.

**Additional file 6.** Random intercept estimates and 95% HDI for 29 countries with more than 1 observations.

## Data Availability

Researcher interested in the data obtained from CE-DAT are requested to contact the Center for Research on the Epidemiology of Disasters.

## Abbreviations

CAR: Central African Republic
CE-DAT: Complex Emergence Database
GAM: Global Acute Malnutrition
HDI: Highest Density Interval
IDPs: Internally Displaced Persons
MCV: Measles Containing Vaccination
MCMC: Markov Chain Monte Carlo
PPC: Posterior Predictive Check
U5DR: Under-five death rate

## References

[1] Development Initiatives. Global Humanitarian Assistance Report 2020. 2020. Available from: https://devinit.org/resources/global-humanitarian-assistance-report-2020/people-and-crisis/#:~:text=Protracted%20crisis%20countries%20are%20defined,of%20Korea%20(PDR%20Korea). Accessed 18 May 2021.

[2] United Nations High Commissioner for Refugees. Global Trends Forced Displacement in 2020. Available from: https://www.unhcr.org/figures-at-a-glance.html. Accessed 29 July 2021.

[3] United Nations High Commissioner for Refugees. Global Trends. Forced Displacement in 2019. 2020. Available from: https://www.unhcr.org/5ee200e37.pdf. Accessed 14 Sept 2021.

[4] Nicholson F, Kumin J. A guide to international refugee protection and building state asylum systems Handbook for Parliamentarians N° 27, 2017. 2017. Inter-Parliamentary Union and the United Nations High Commissioner for Refugees. Available from: https://www.unhcr.org/publications/legal/3d4aba564/refugee-protection-guide-international-refugee-law-handbook-parliamentarians.html. Accessed 29 July 2021

[5] European Union, United Nations. Expert Group on Refugee and Internally Displaced Persons Statistics. International Recommendations on Refugee Statistics. 2018. Available from: https://unstats.un.org/unsd/demographic-social/Standards-and-Methods/files/Principles_and_Recommendations/International-Migration/2018_1746_EN_08-E.pdf. Accessed 29 July 2021.

[6] United Nations International Children's Emergency Fund. Child displacement. 2021. Available from: https://data.unicef.org/topic/child-migration-and-displacement/displacement/. Accessed 29 July 2021.

[7] Moore PS, Marfin AA, Quenemoen LE, Gessner BD, Ayub YS, Miller DS (1993). Mortality rates in displaced and resident populations of central Somalia during 1992 famine. Lancet.

[8] Heudtlass P, Speybroeck N, Guha-Sapir D (2016). Excess mortality in refugees, internally displaced persons and resident populations in complex humanitarian emergencies (1998–2012) – insights from operational data. Confl Heal.

[9] Action Against Hunger. Integrated nutrition and mortality SMART survey report: Hudur Town, Bakool Region, Somalia. 2020. Available from: https://www.humanitarianresponse.info/sites/www.humanitarianresponse.info/files/assessments/smart_survey_repor_acf_hudur_district_october_2020_1.pdf. Accessed 27 Aug 2021.

[10] . UNHCR, WFP, ARRA, IMC, MSF-S, Humedica, et al. Standardized expanded nutrition survey in Melkadid refugee camps. 2018. Available from: https://reliefweb.int/sites/reliefweb.int/files/resources/66696.pdf. Accessed 27 Aug 2021.

[11] World Health Organization, Sudan FMoH. Mortality survey among Internally Displaced Persons and other affected populations in Greater Darfur, Sudan. 2005. Available from: https://reliefweb.int/sites/reliefweb.int/files/resources/DE2FAE55979C73634925709E00085B3F-who-sdn-30sep.pdf. Accessed 29 July 2021.

[12] Blössner M, De Onis M, Prüss-Üstün A (2005). Malnutrition : quantifying the health impact at national and local levels / Monika Blössner and Mercedes de Onis.

[13] Delbiso TD, Altare C, Rodriguez-Llanes JM, Doocy S, Guha-Sapir D (2017). Drought and child mortality: a meta-analysis of small-scale surveys from Ethiopia. Sci Rep.

[14] Mason JB, White JM, Heron L, Carter J, Wilkinson C, Spiegel P (2012). Child acute malnutrition and mortality in populations affected by displacement in the Horn of Africa, 1997–2009. Int J Environ Res Public Health.

[15] Guerrier G, Zounoun M, Delarosa O, Defourny I, Lacharite M, Brown V (2009). Malnutrition and mortality patterns among internally displaced and non-displaced population living in a camp, a village or a town in Eastern Chad. PloS One.

[16] Olwedo MA, Mworozi E, Bachou H, Orach CG (2008). Factors associated with malnutrition among children in internally displaced person's camps, northern Uganda. Afr Health Sci.

[17] Leidman E, Tromble E, Yermina A, Johnston R, Isokpunwu C, Adeniran A (2017). Acute malnutrition among children, mortality, and humanitarian interventions in conflict-affected regions - Nigeria, October 2016-March 2017. MMWR Morb Mortal Wkly Rep.

[18] Oluwatosin AB, Tosin A, Udo EM (2019). Malnutrition among internally displaced persons children: a consequence of armed conflicts in Nigeria. J Glob Peace Confl.

[19] Connolly MA, Gayer M, Ryan MJ, Salama P, Spiegel P, Heymann DL (2004). Communicable diseases in complex emergencies: impact and challenges. Lancet.

[20] Seal AJ, Jelle M, Grijalva-Eternod CS, Mohamed H, Ali R, Fottrell E (2021). Use of verbal autopsy for establishing causes of child mortality in camps for internally displaced people in Mogadishu, Somalia: a population-based, prospective, cohort study. Lancet Glob Health.

[21] Torbosh A, Al Amad MA, Al Serouri A, Khader Y (2019). The impact of war in Yemen on immunization coverage of children under one year of age: descriptive study. JMIR Public Health Surveill.

[22] Kouadio IK, Koffi AK, Attoh-Toure H, Kamigaki T, Oshitani H (2009). Outbreak of measles and rubella in refugee transit camps. Epidemiol Infect.

[23] Musa TH, Kambo RI, Ahmed AA, Musa HH (2017). Epidemiology of measles cases in south Darfur State, Sudan, 2011–2015. Biomed Environ Sci.

[24] Roberton T, Weiss W, Doocy S. Challenges in Estimating Vaccine Coverage in Refugee and Displaced Populations: Results From Household Surveys in Jordan and Lebanon. Vaccines (Basel). 2017;5(3). 10.3390/vaccines5030022 PMC5620553 28805672

[25] Desai AN, Ramatowski JW, Marano N, Madoff LC, Lassmann B (2020). Infectious disease outbreaks among forcibly displaced persons: an analysis of ProMED reports 1996–2016. Confl Health.

[26] Jean Baptiste AE, Wagai J, Luce R, Masresha B, Klinkenberg D, Veldhuijzen I (2021). Measles outbreak in complex emergency: estimating vaccine effectiveness and evaluation of the vaccination campaign in Borno State, Nigeria, 2019. BMC Public Health.

[27] Korave J, Bawa S, Ageda B, Ucho A, Bem-Bura DM, Onimisi A (2021). Internal displacement; an impediment to the successful implementation of planned measles supplemental activities in Nigeria, a case study of Benue State. Vaccine.

[28] Severity of Measles in Malnutrition. Nutr Rev. 1982;40(7):203-205. 10.1111/j.1753-4887.1982.tb05310.x. 10.1111/j.1753-4887.1982.tb05310.x 6181440

[29] World Health Organization, Refugee and migrant health [Fact sheet], May 2022. Available from:https://www.who.int/news-room/fact-sheets/detail/refugee-and-migrant-health. Accessed 07 June 2022.

[30] Navarro-Colorado C, Mahamud A, Burton A, Haskew C, Maina GK, Wagacha JB (2014). Measles outbreak response among adolescent and adult Somali refugees displaced by famine in Kenya and Ethiopia, 2011. J Infect Dis.

[31] Kamugisha C, Cairns KL, Akim C. An Outbreak of Measles in Tanzanian Refugee Camps. J Infect Dis. 2003;187(1):S58–62. 10.1086/368057 12721892

[32] Sphere Project (2011). Sphere Handbook: Humanitarian Charter and Minimum Standards in Disaster Response.

[33] Weissman F. Mortality emergency threshold: A case for revision [press release]. ALNAP. 2018.

[34] SMART, Measuring Mortality, Nutritional Status and Food Security in Crisis Situations: The SMART Protocol. Version 1. 2005. Available from: https://www.unhcr.org/45f6b2c42.pdf. Accessed 20 Nov 2021

[35] Altare C, Guha-Sapir D (2014). The complex emergency database: a global repository of small-scale surveys on nutrition, health and mortality. PLoS One.

[36] The United Nations Inter-agency Group for Child Mortality Estimation. 2021. Available from: https://childmortality.org/data. Accessed 03 May 2022.

[37] The United Nations High Commissioner for Refugees. UNHCR Emergency Handbook. Acute Malnutrition threshold. 2015. Available from: https://emergency.unhcr.org/entry/32604/acute-malnutrition-threshold. Accessed 06 August 2021.

[38] Cutts FT, Izurieta HS, Rhoda DA (2013). Measuring coverage in MNCH: design, implementation, and interpretation challenges associated with tracking vaccination coverage using household surveys. PLoS Med.

[39] Brown VA (2021). An introduction to linear mixed-effects modeling in R. Adv Methods Pract Psychol Sci.

[40] Lavine M (1999). What is Bayesian statistics and why everything else is wrong. J Undergrad Math Applications.

[41] John KK. Doing Bayesian Data Analysis: A tutorial with R, JAGS, and Stan. 2nd ed. Amsterdam: Academic Press; 2014.

[42] Gelman A, Stern HS, Carlin JB, Rubin DB (2014). Bayesian Data Analysis.

[43] Plummer M (2019). rjags: Bayesian Graphical Models using MCMC.

[44] Meredith M, Kruschke J (2020). HDInterval: Highest (Posterior) Density Intervals.

[45] Wickham H (2016). ggplot2: Elegant Graphics for Data Analysis.

[46] Abdullahi SA, Smelyanskaya M, John S, Adamu HI, Ubochioma E, Kennedy I (2020). Providing TB and HIV outreach services to internally displaced populations in Northeast Nigeria: Results of a controlled intervention study. PLoS Med.

[47] Alix-Garcia J, Bartlett A, Saah D (2013). The landscape of conflict: IDPs, aid and land-use change in Darfur. J Econ Geogr.

[48] Alix-Garcia J, Walker S, Bartlett A, Onder H, Sanghi A (2018). Do refugee camps help or hurt hosts? The case of Kakuma, Kenya. J Dev Econ.

[49] Alix-Garcia J, Saah D (2009). The effect of refugee inflows on host communities: evidence from Tanzania. World Bank Econ Rev.

[50] Campbell AA, de Pee S, Sun K, Kraemer K, Thorne-Lyman A, Moench-Pfanner R (2009). Relationship of household food insecurity to neonatal, infant, and under-five child mortality among families in rural Indonesia. Food Nutr Bull.

[51] Asiseh F, Naanwaab C, Quaicoe O (2018). The association between food insecurity and child health outcomes in low and middle-income countries. Journal of African Development.

[52] Goldhaber-Fiebert JD, Lipsitch M, Mahal A, Zaslavsky AM, Salomon JA (2010). Quantifying child mortality reductions related to measles vaccination. PLoS One.

[53] Van den Ent MM, Brown DW, Hoekstra EJ, Christie A, Cochi SL (2011). Measles mortality reduction contributes substantially to reduction of all cause mortality among children less than five years of age, 1990–2008. J Infect Dis.

[54] McGovern ME, Canning D (2015). Vaccination and all-cause child mortality from 1985 to 2011: global evidence from the demographic and health surveys. Am J Epidemiol.

[55] Kett M (2005). Displaced populations and long term humanitarian assistance. BMJ.

[56] Singh K, Karunakara U, Burnham G, Hill K (2005). Forced migration and under-five mortality: a comparison of refugees and hosts in North-Western Uganda and Southern Sudan. J Popul Rev Européenne de Démographie.

