# Supplementary material for: Predictors of exceeding emergency under-five mortality thresholds using small-scale survey data from humanitarian settings (1999 – 2020): considerations for measles vaccination, malnutrition, and displacement status
Source: Arch Public Health. 2022 Jun 28;80:160. doi: 10.1186/s13690-022-00916-0 (PMC9238088; doi:10.1186/s13690-022-00916-0)
Supplement: Supplementary file 1 — Additional file 1. R code for Bayesian multivariable Fixed and Mixed-Effect logistic regression. [file 13690_2022_916_MOESM1_ESM.docx]

Additional file 1: RCode of Bayesian fixed and mixed-effect logic regression model

############################## Bayesian multivariable fixed-effect logistic regression ####################

"model{

for(i in 1:N){

new_u5dr[i] ~ dbern(mu[i])

logit(mu[i]) <- delta[1] + delta[2]*mcv[i] + delta[3]*gam[i] + delta[4]*popstat_rfg[i] + delta[5]*popstat_rsd[i]

## popstat_rfg indicator variable for refugees

## popstat_rsd indicator variable for affected residents

#########

new.u5dr[i] ~ dbern(mu[i]) ## generate new values

var.new_u5dr[i] <- mu[i]*(1-mu[i]) ## variance of observed values

pred.res[i] <- (new_u5dr[i] - mu[i])/sqrt(var.new_u5dr[i])

pred.new.res[i] <- (new.u5dr[i] - mu[i])/sqrt(var.new_u5dr[i])

Dv[i] <- pow(pred.res[i],2)

Dv.new[i] <- pow(pred.new.res[i],2)

###

}

for(j in 1:5){

delta[j] ~ dnorm(0,1.0e-6) ## assign non-informative normal prior

}

fit <- sum(Dv[])

fit.new <- sum(Dv.new[])

}"

################################################## END#####################

################## Bayesian multivariable mixed-effect logistic regression #################

"model{

for(i in 1:N){

new_u5dr[i] ~ dbern(mu[i])

logit(mu[i]) <- delta[1] + delta[2]*mcv[i] + delta[3]*gam[i] + delta[4]*popstat_rfg[i] +delta[5]*popstat_rsd[i] + r[country[i]]

#########

new.u5dr[i] ~ dbern(mu[i]) ## generate new values

var.new_u5dr[i] <- mu[i]*(1-mu[i]) ## variance of observed values

pred.res[i] <- (new_u5dr[i] - mu[i])/sqrt(var.new_u5dr[i])

pred.new.res[i] <- (new.u5dr[i] - mu[i])/sqrt(var.new_u5dr[i])

Dv[i] <- pow(pred.res[i],2)

Dv.new[i] <- pow(pred.new.res[i],2)

}

r[nk] <- 0 ## set observations with single value to 0

for(k in 1:ncntry){ ## ncntry number of countries with more than 1 observation

r[k] ~ dnorm(0,sige) ## assign random variable to countries with more than 1 observation

}

taue ~ dgamma(0.001,0.001)

sige <- pow(taue,-2)

for(j in 1:5){ # 5 is the number of parameters being estimated

delta[j] ~ dnorm(0,1.0e-6) ## non-informative prior to model parameters

}

fit <- sum(Dv[])

fit.new <- sum(Dv.new[])

}"

################# END ##########################################################
