# Supplementary material for: Predictors of exceeding emergency under-five mortality thresholds using small-scale survey data from humanitarian settings (1999 – 2020): considerations for measles vaccination, malnutrition, and displacement status
Source: Arch Public Health. 2022 Jun 28;80:160. doi: 10.1186/s13690-022-00916-0 (PMC9238088; doi:10.1186/s13690-022-00916-0)
Supplement: Supplementary file 2 — Additional file 2. Summary of the surveys (n=1597) from 1999 to 2020. [file 13690_2022_916_MOESM2_ESM.docx]

Additional file 2: Summary of the surveys (n=1597) from 1999 to 2020

| **country** | **N.surveys** | **No.years** | **Tot.sample** | **No.surveys** | **IDP** | **Refugee** | **Resident** |
| --- | --- | --- | --- | --- | --- | --- | --- |
| Afghanistan | 21 | 8 | 6021 | 6 | 4 | 0 | 17 |
| Angola | 31 | 5 | 23244 | 17 | 18 | 0 | 13 |
| Bangladesh | 12 | 5 | 4660 | 7 | 0 | 5 | 7 |
| Burkina Faso | 1 | 1 | 3126 | 1 | 0 | 0 | 1 |
| Burundi | 12 | 7 | 6506 | 8 | 0 | 0 | 12 |
| Cameroon | 1 | 1 | NA | NA | 0 | 1 | 0 |
| Central African Republic | 10 | 4 | 5256 | 5 | 0 | 0 | 10 |
| Chad | 72 | 7 | 26634 | 22 | 3 | 52 | 17 |
| Cote d'Ivoire | 1 | 1 | 1278 | 1 | 0 | 0 | 1 |
| Democratic Republic of the Congo | 284 | 17 | 88581 | 82 | 1 | 3 | 280 |
| Djibouti | 1 | 1 | 399 | 1 | 0 | 0 | 1 |
| Ethiopia | 377 | 18 | 17740 | 22 | 4 | 82 | 291 |
| Ghana | 2 | 2 | NA | NA | 0 | 2 | 0 |
| Guatemala | 1 | 1 | 1555 | 1 | 0 | 0 | 1 |
| Guinea | 6 | 4 | 2186 | 3 | 0 | 0 | 6 |
| Haiti | 41 | 6 | 5425 | 11 | 0 | 0 | 41 |
| Kenya | 71 | 13 | 44495 | 44 | 0 | 9 | 62 |
| Liberia | 18 | 4 | 10628 | 9 | 13 | 0 | 5 |
| Malawi | 8 | 5 | 9056 | 19 | 0 | 0 | 8 |
| Mali | 18 | 7 | 1674 | 1 | 0 | 0 | 18 |
| Mauritania | 9 | 4 | 19332 | 13 | 0 | 1 | 8 |
| Myanmar | 3 | 2 | 8087 | 9 | 0 | 0 | 3 |
| Niger | 55 | 6 | 24732 | 36 | 0 | 0 | 55 |
| Nigeria | 1 | 1 | 1633 | 1 | 0 | 0 | 1 |
| Occupied Palestinian Territories | 3 | 1 | NA | NA | 0 | 0 | 3 |
| Pakistan | 10 | 5 | NA | NA | 0 | 4 | 6 |
| Philippines | 5 | 2 | NA | NA | 0 | 0 | 5 |
| Sierra Leone | 22 | 9 | 20683 | 9 | 14 | 0 | 8 |
| Somalia | 174 | 10 | 17403 | 13 | 36 | 0 | 138 |
| South Sudan | 19 | 4 | 11693 | 15 | 0 | 8 | 11 |
| Sudan | 222 | 14 | 81022 | 73 | 80 | 6 | 136 |
| Syrian Arab Republic | 1 | 1 | NA | NA | 0 | 1 | 0 |
| Tajikistan | 7 | 2 | 920 | 1 | 0 | 0 | 7 |
| Timor-Leste | 1 | 1 | 1185 | 1 | 0 | 0 | 1 |
| Uganda | 23 | 5 | 7046 | 19 | 12 | 0 | 11 |
| Yemen | 33 | 6 | 20407 | 25 | 1 | 3 | 29 |
| Zimbabwe | 21 | 5 | 19100 | 1 | 0 | 0 | 21 |

NB: Tot.sample is the number of children samples in mortality surveys. While No.surveys is the number of surveys that reported the number of children included for the mortality surveys.
