# Supplementary material for: Predictors of exceeding emergency under-five mortality thresholds using small-scale survey data from humanitarian settings (1999 – 2020): considerations for measles vaccination, malnutrition, and displacement status
Source: Arch Public Health. 2022 Jun 28;80:160. doi: 10.1186/s13690-022-00916-0 (PMC9238088; doi:10.1186/s13690-022-00916-0)
Supplement: Supplementary file 3 — Additional file 3: Figure 1. Traceplots of mixed-effect model III. Figure 2. Traceplots of mixed-effect model IV. [file 13690_2022_916_MOESM3_ESM.docx]

Figure 1: Traceplots of mixed-effect model III


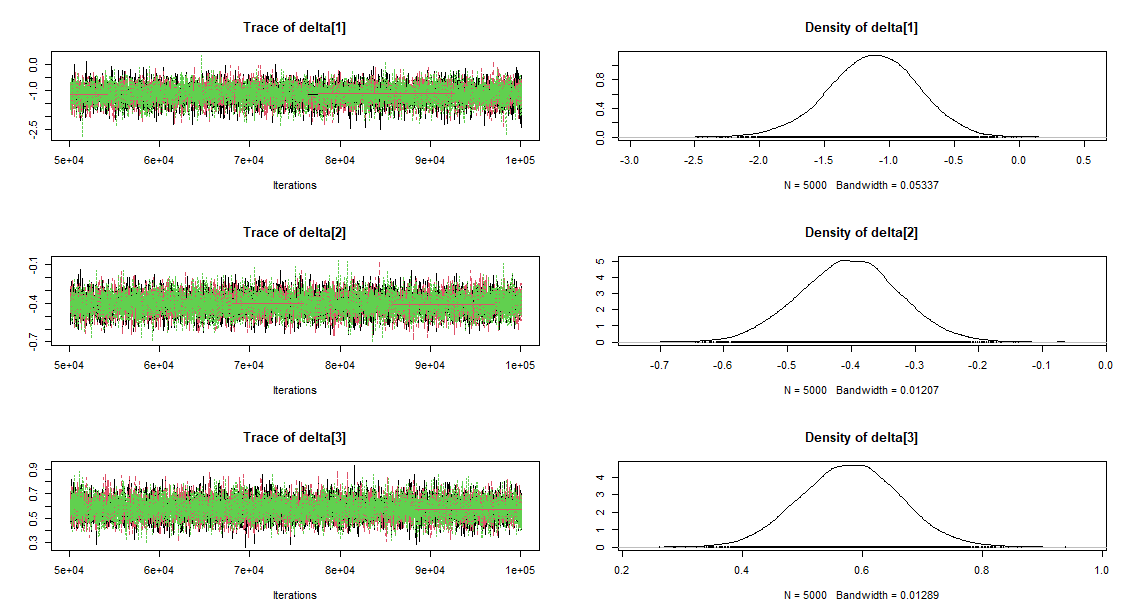

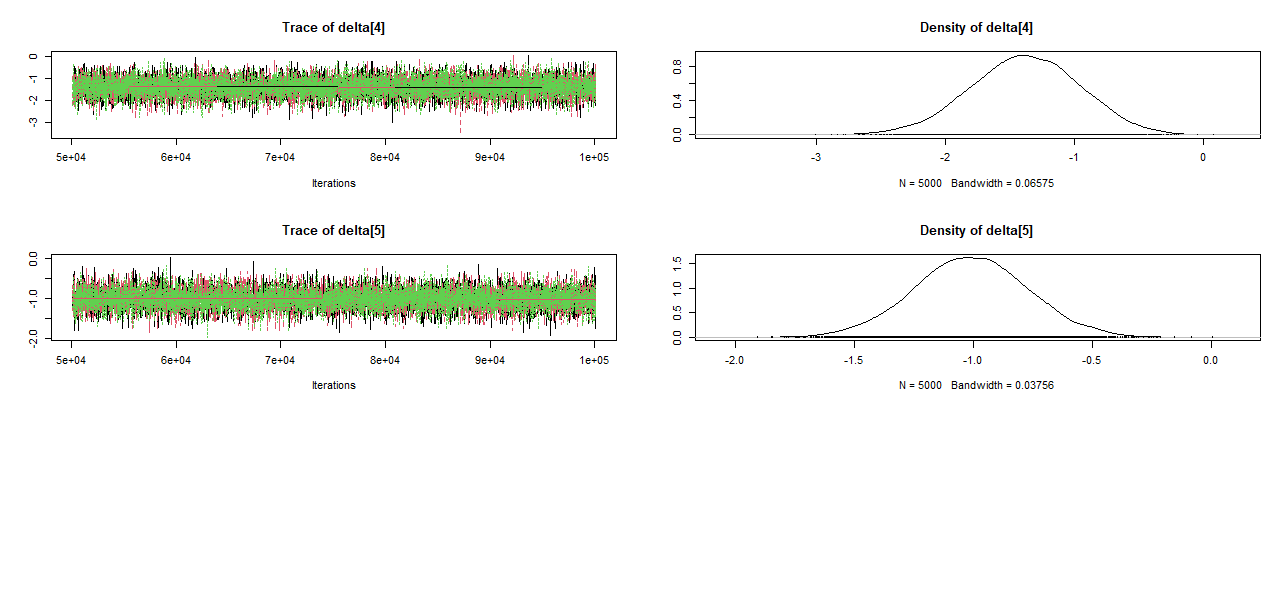


delta[1], delta[2], delta[3], delta[4] and delta[5] are the intercept, measles vaccination coverage, prevalence of global acute malnutrition, Refugees and resident-affected coefficients.

Figure 2: Traceplots of mixed-effect model IV


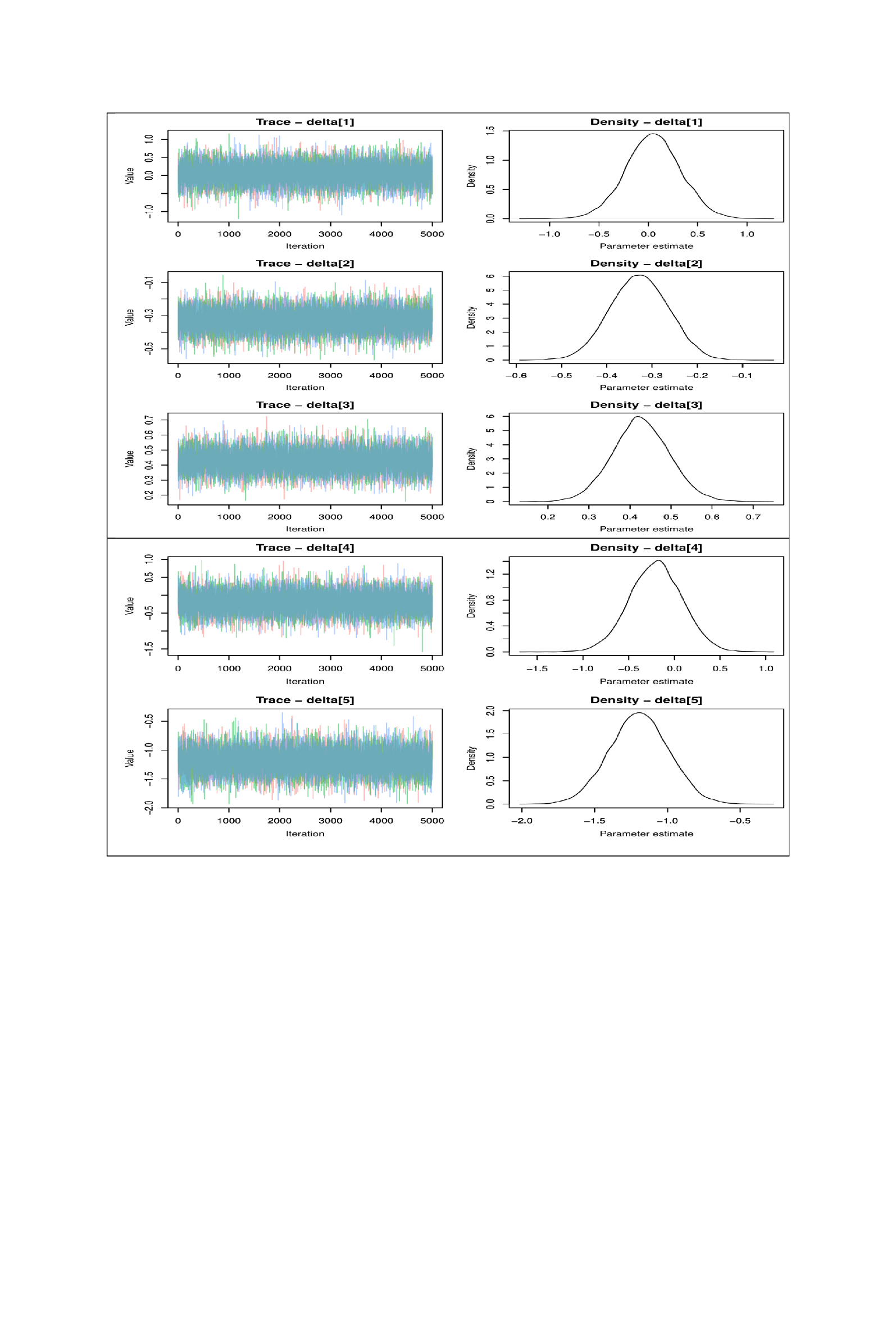


delta[1], delta[2], delta[3], delta[4] and delta[5] are the intercept, measles vaccination coverage, prevalence of global acute malnutrition, Refugees and resident-affected coefficients.
