# Supplementary material for: Predictors of exceeding emergency under-five mortality thresholds using small-scale survey data from humanitarian settings (1999 – 2020): considerations for measles vaccination, malnutrition, and displacement status
Source: Arch Public Health. 2022 Jun 28;80:160. doi: 10.1186/s13690-022-00916-0 (PMC9238088; doi:10.1186/s13690-022-00916-0)

Additional file 4.1: posterior predictive check for Bayesian multivariable fixed-effect logistic regression model I


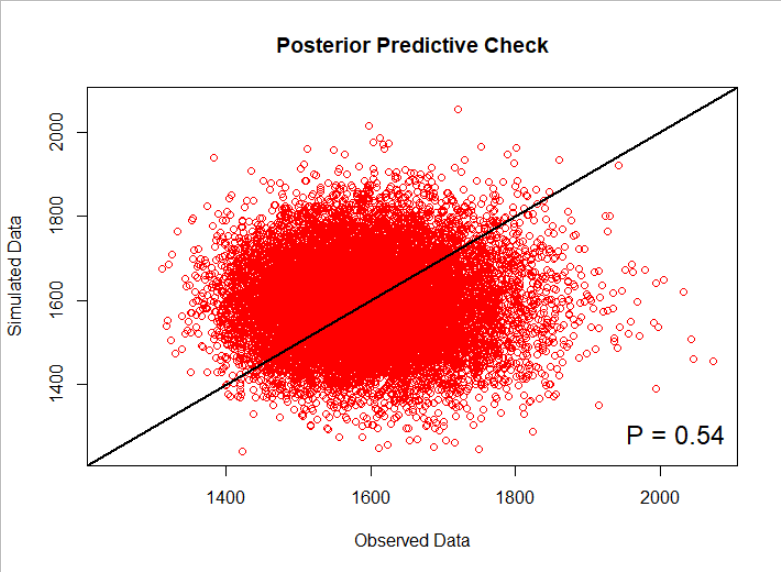


Additional file 4.2: posterior predictive check for Bayesian multivariable fixed-effect logistic regression model II


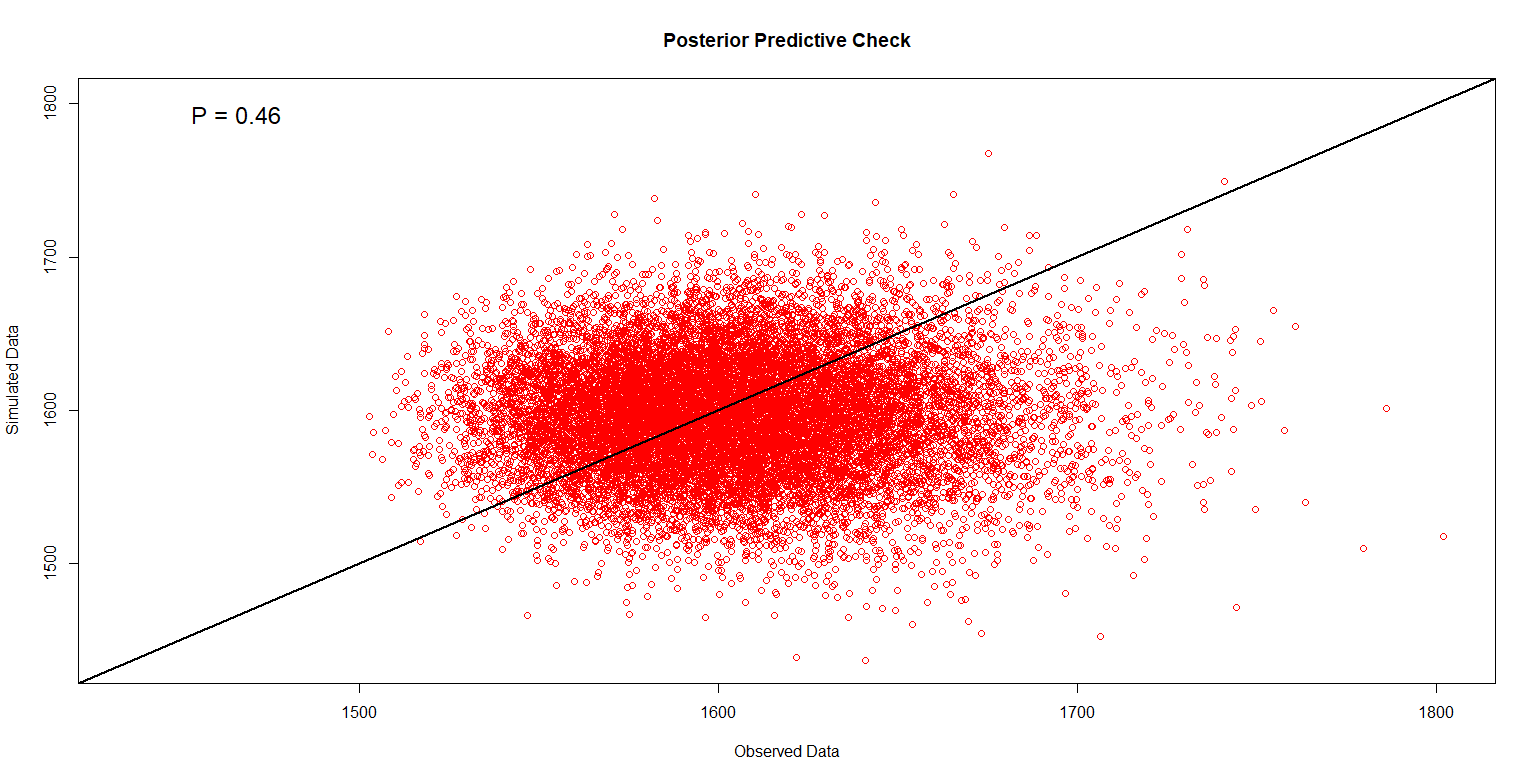

Supplement: Supplementary file 4 — Additional file 4. PPC check for Bayesian multivariable mixed-effect logistic regression model. [file 13690_2022_916_MOESM4_ESM.docx]
