# Supplementary material for: Predictors of exceeding emergency under-five mortality thresholds using small-scale survey data from humanitarian settings (1999 – 2020): considerations for measles vaccination, malnutrition, and displacement status
Source: Arch Public Health. 2022 Jun 28;80:160. doi: 10.1186/s13690-022-00916-0 (PMC9238088; doi:10.1186/s13690-022-00916-0)
Supplement: Supplementary file 6 — Additional file 6. Random intercept estimates and 95% HDI for 29 countries with more than 1 observations. [file 13690_2022_916_MOESM6_ESM.docx]

Additional file 6: Random intercept estimates and 95% HDI for 29 countries with more than 1 observations

|  | **Model III** | | | **Model IV** | | |  |
| --- | --- | --- | --- | --- | --- | --- | --- |
| **Parameter** | **mean** | **2.50%** | **97.50%** | **mean** | **2.5%** | **97.5%** | **country** |
| r[1] | 0.76 | -0.38 | 1.88 | 0.302 | -0.638 | 1.209 | Afghanistan |
| r[2] | 3.07 | 2.04 | 4.21 | 1.50 | 0.655 | 2.411 | Angola |
| r[3] | -0.29 | -1.99 | 1.18 | -0.42 | -1.62 | 0.69 | Bangladesh |
| r[4] | 0.77 | -0.79 | 2.23 | 1.007 | -0.106 | 2.133 | Burundi |
| r[5] | 0.08 | -1.67 | 1.65 | -0.41 | -1.88 | 0.864 | Central African Republic |
| r[6] | -0.68 | -1.79 | 0.33 | -1.548 | -2.408 | -0.765 | Chad |
| r[7] | 0.79 | 0.17 | 1.48 | 1.097 | 0.617 | 1.602 | Democratic Republic of the Congo |
| r[8] | -0.32 | -0.97 | 0.36 | -0.164 | -0.659 | 0.329 | Ethiopia |
| r[9] | -0.21 | -2.59 | 1.90 | -0.576 | -2.423 | 1.039 | Ghana |
| r[10] | 0.17 | -1.63 | 1.81 | 0.248 | -1.155 | 1.608 | Guinea |
| r[11] | -1.41 | -3.41 | 0.07 | -1.089 | -2.262 | -0.087 | Haiti |
| r[12] | 0.33 | -0.48 | 1.17 | 1.387 | 0.758 | 2.04 | Kenya |
| r[13] | 1.39 | 0.31 | 2.53 | 0.718 | -0.246 | 1.696 | Liberia |
| r[14] | -0.50 | -2.69 | 1.32 | -0.156 | -1.668 | 1.203 | Malawi |
| r[15] | -1.26 | -3.29 | 0.27 | -1.488 | -3.138 | -0.229 | Mali |
| r[16] | -0.78 | -2.93 | 0.92 | -0.125 | -1.457 | 1.11 | Mauritania |
| r[17] | -0.68 | -2.86 | 1.16 | -0.739 | -2.519 | 0.777 | Myanmar |
| r[18] | 0.74 | -0.07 | 1.57 | 0.545 | -0.116 | 1.221 | Niger |
| r[19] | -0.20 | -2.56 | 1.93 | -0.306 | -2.218 | 1.364 | Occupied Palestinian Territories |
| r[20] | -1.24 | -3.32 | 0.37 | -1.044 | -2.48 | 0.192 | Pakistan |
| r[21] | -0.55 | -2.80 | 1.30 | 0.444 | -0.978 | 1.846 | Philippines |
| r[22] | 0.13 | -1.05 | 1.27 | -0.742 | -1.825 | 0.217 | Sierra Leone |
| r[23] | 0.08 | -0.56 | 0.78 | -0.445 | -1.003 | 0.103 | Somalia |
| r[24] | 0.28 | -0.93 | 1.43 | -0.462 | -1.472 | 0.498 | South Sudan |
| r[25] | -0.81 | -1.50 | -0.09 | 0.253 | -0.262 | 0.775 | Sudan |
| r[26] | -0.51 | -2.71 | 1.32 | -0.706 | -2.46 | 0.728 | Tajikistan |
| r[27] | -0.28 | -1.65 | 0.97 | 0.055 | -0.894 | 0.959 | Uganda |
| r[28] | -0.58 | -1.95 | 0.59 | -0.114 | -0.972 | 0.694 | Yemen |
| r[29] | -0.29 | -1.94 | 1.14 | 0.073 | -0.993 | 1.06 | Zimbabwe |
| sigma | 0.83 | 0.31 | 1.78 | 0.99 | 0.66 | 1.46 |  |
| tau | 1.18 | 0.75 | 1.80 | 1.16 | 0.47 | 2.33 |  |
